# Role of Confined Optical Phonons in Exciton Generation in Spherical Quantum Dot

**DOI:** 10.3390/ma15165545

**Published:** 2022-08-12

**Authors:** Ramji Singh, Mitra Dutta, Michael A. Stroscio

**Affiliations:** 1 Department of Electrical and Computer Engineering, University of Illinois at Chicago, 851 S Morgan Street, Chicago, IL 60607, USA; 2 Department of Physics, University of Illinois at Chicago, 845 W Taylor Street, Chicago, IL 60607, USA; 3 Department of Bioengineering, University of Illinois at Chicago, 851 S Morgan Street, Chicago, IL 60607, USA

**Keywords:** confined LO phonon, dressed states, exciton, quantum dot, qubit

## Abstract

Optical control of excitonic states in semiconducting quantum dots has enabled it to be deployed as a qubit for quantum information processing. For self-assembled quantum dots, these excitonic states couple with phonons in the barrier material, for which the previous studies have shown that such exciton—phonon coupling can also lead to the generation of exciton, paving the way for their deployment in qubit-state preparation. Previous studies on self-assembled quantum dots comprising polar materials have considered exciton—phonon coupling by treating phonon modes as bulk acoustic modes only, owing to nearly the same acoustic property of the dot and barrier material. However, the dimensional confinement leads to significant modification phonon modes, even though acoustic confinement is weak but optical confinement cannot be overlooked. In this paper, we investigate for the first time the exciton—optical phonon coupling using dielectric continuum model duly accounting for the dimensional confinement leading to exciton generation. We report that at low temperatures (below 10 K), the exciton creation rate attributed to confined optical phonon is approximately 5.7 times (~6) slower than bulk acoustic phonons, which cannot be ignored, and it should be accounted for in determining the effective phonon assisted exciton creation rate.

## 1. Introduction

The discrete energy levels in quantum dots (QDs) makes them attractive in quantum information processing for applications such as single photon source [1] and entangled photon source [2] due to the confinement of carriers in three dimensions. The excitonic excitations in a single QD form a basis for a two-level system to be implemented as a qubit in quantum computing applications [3]. The qubit states can be manipulated by optical excitations using laser sources [4]. In the recent past, numerous studies have been done with III-V-based self-assembled QDs for qubit state preparation [5,6,7,8,9]. These self-assembled quantum dots are nanoscale islands of a smaller bandgap material embedded in the matrix of larger bandgap material with a smaller mismatch in their elastic properties [10]. The discrete energy states in the QD are subjected to interactions with phonon modes, leading to dephasing [9,10,11]. However, the phonon modes also lead to the creation of excitons and such schemes have been studied extensively by treating the phonons as bulk acoustic phonon modes [5,6,7,8,9]. However, in the past, experimental observation was conducted on the role of longitudinal optical (LO) phonon-assisted exciton generation in InGaAs/GaAs based QD [12]; it was observed that the exciton generation was caused due to the emission of LO phonon frequencies which are equal to the near-zone center (in GaAs) value. We would like to point out that with the dimensional confinement, as in III-V based QDs, the polar optical phonon modes undergo significant modification [13,14,15]. Such modifications of polar optical phonons have been studied previously under the purview of the dielectric continuum model, which assumes that the associate lattice vibrations of a material produce a macroscopic polarization P(r) which is describable in terms of the equations of electrostatics of a medium with dielectric constant ε(ω) [14]. These equations, when subjected to boundary conditions, depending on the geometry of the medium, yields the phonon modes and the associated Fröhlich potential which interacts (perturbs) the charge carriers. To properly treat polar optical phonon—exciton interaction in a quantum dot, it is essential to consider such modified optical phonon modes instead of bulk optical modes. The modified polar optical modes are (a) confined LO modes, and (b) surface optical (SO) modes. The confined optical phonon-assisted creation of exciton in the presence of laser light has been overlooked in the existing literature. In this paper we investigate the role of confined LO phonon modes using the dielectric continuum model [14,16,17] in the creation of excitons when subjected to a continuous optical energy from an incident laser. We consider a GaAs spherical quantum dot of radius 3.39 nm (12 monolayer thickness) as a representative of a strongly confined quantum dot.

## 2. Materials and Methods

### 2.1. Description of Confined Optical Modes

Let the quantum dot of radius R with a dielectric constant $\varepsilon_{1}\left(\omega \right)$ be embedded in the barrier material with dielectric constant $\varepsilon_{2}\left(\omega \right)$. The polar optical phonon modes inside and outside the quantum dot produce macroscopic polarization ***P***, due to which the associated electric displacement vector ***D***, the electric field ***E*,** and the Fröhlich potential, Φ, in each medium are related as follows [14,16,17]:

(1) D=εE=E+4πP

(2) E=−∇Φ 

(3) ∇⋅D=0 

In Equation (1), ε(ω) is obeys the Lyddane-Sach-Teller relation as below:

(4) $$\varepsilon_{1,2}\left(\omega \right)=\varepsilon_{\infty }\frac{\omega^{2}-\omega_{LO_{1,2}}^{2}}{\omega^{2}-\omega_{TO_{1,2}}^{2}}$$

where, $\omega_{LO}$ and $\omega_{TO}$ are the zone center longitudinally optical (LO) and transverse optical (TO) phonon frequency and $\varepsilon_{\infty }$ is the high frequency dielectric constant. The divergence of the displacement vector ***D*** vanishes in Equation (3) because it is assumed that no free charge exists inside the material. Now, from Equations (1)–(3), we get the following relation in each medium:

(5) $$\varepsilon \left(\omega \right)\nabla^{2}\Phi \left(r\right)=0$$

There are two possible solutions which satisfy Equation (4): (a) confined LO phonon modes, which corresponds to ε(ω)=0 (b) surface optical modes, which corresponds to $\nabla^{2}\Phi \left(r\right)=0$. For the confined LO modes, the phonon frequency satisfies ε(ω)=0, which results in $\omega =\omega_{LO}$ and the eigenfunction corresponding to potential Φ(r) can be expressed in spherical coordinates (with origin at the center of the quantum dot) after expanding in terms of complete set of orthogonal functions $j_{l}\left(qr\right)Y_{l}^{m}\left(\theta ,\varphi \right)$ as follows:

(6) $$\Phi \left(r\right)=\underset{l,m}{\sum }\underset{q}{\sum }B_{q}j_{l}\left(qr\right)Y_{l}^{m}\left(\theta ,\varphi \right)$$

In Equation (6), $j_{l}\left(qr\right)$ with l=0, 1, 2, 3… represents the spherical Bessel functions of order l, $Y_{l}^{m}\left(\theta ,\varphi \right)$ represents the spherical harmonics such that −l≤m≤l and $B_{q}=\sqrt{\frac{2}{R^{3}}}\cdot \frac{1}{j_{l+1}\left(qR\right)}$ is the normalization constant. Using Equation (6), after imposing the electrostatic boundary conditions: (a) continuity of the potential Φ at the interface (b) continuity of normal component of the displacement vector ***D***, we see that the potential Φ must vanish at the interface r=R; hence, equating Equation (6) to zero we get:

(7) $$j_{l}\left(qR\right)=0$$

Equation (7) leads to the following solution:

(8) $$q_{n}=\frac{x_{n,l}}{R}$$

where, $x_{n,l}$ is the nth zero of the lth order spherical Bessel function. Equation (8) is very significant as it describes the effect of dimensional confinement on phonon wave vector, which becomes discrete as opposed to being continuous in the case of bulk material. Finally, the Fröhlich potential as given in Equation (6) can be represented in second quantized form after duly considering quantization of amplitudes of the ionic pair of the material as below [13]:

(9a) $$\Phi \left(r\right)=\underset{l,m}{\sum }\underset{n}{\sum }f_{lm}\left(q_{n}\right)\left[{\hat{a}}_{l,m}\left(q_{n}\right)j_{l}\left(q_{n}r\right)Y_{l}^{m}\left(\theta ,\varphi \right)+H.C.\right]$$

where,

(9b) $$f_{lm}\left(q_{n}\right)={\left(\frac{2\pi \hbar \omega_{LO}B_{q}^{2}}{q_{n}^{2}}\right)}^{1/2}{\left(\frac{1}{\epsilon_{\infty }}-\frac{1}{\epsilon_{0}}\;\right)}^{1/2}$$

In Equation (9a), H.C. represents Hermitian conjugate and ${\hat{a}}_{l,m}$ is the phonon annihilation operator.

### 2.2. Confined LO Phonon—Exciton Interaction 

We assume a parabolic confinement potential for the exciton in the ground state [9], so that the net charge density can be written as:

(10) $$\rho \left(r\right)=-e\left(\frac{e^{-r^{2}/a_{e}^{2}}}{\pi^{3/2}a_{e}^{3}}-\frac{e^{-r^{2}/a_{h}^{2}}}{\pi^{3/2}a_{h}^{3}}\right)$$

where, $a_{e}$ and $a_{h}$ are the electron and hole confinement length, such that $a_{h}=a_{e}/1.15$ in GaAs taking into account the heavier hole effective mass than that for the electron [9]. The charge density in Equation (10) represents a hole in the heavy hole valence band and electron in the conduction band both with anti-parallel spins. Now, the confined LO phonon mode interacts with the exciton due to the Fröhlich potential Φ, so the interaction Hamiltonian can be expressed as:

(11) $$H_{Fr}=\int^{\;}d^{3}r\;\rho \left(r\right)\;\Phi \left(r\right)\left|2><2\right|$$

Substituting, for Φ(r) from Equation (9a) in Equation (11) we get:

(12) $$H_{Fr}=\underset{l,m}{\sum }\underset{n}{\sum }\left\{{\hat{a}}_{l,m}\left(q_{n}\right)f_{lm}\left(q_{n}\right)\int^{\;}d^{3}r\;j_{l}\left(q_{n}r\right)\;Y_{l}^{m}\left(\theta ,\varphi \right)\;\rho \left(r\right)+H.C\right\}|2><2|$$

Now since,

(13) $$\int_{\varphi =0}^{\varphi =2\pi }\int_{\theta =0}^{\theta =\pi }\;Y_{l}^{m}\left(\theta ,\varphi \right)\sin\theta d\theta \;d\varphi =\sqrt{4\pi }\;\delta_{l,0}\delta_{m,0}$$

Hence, only confined LO phonon modes corresponding to l=0, m=0 mode contribute to the exciton—phonon interaction as represented by the interaction Hamiltonian in Equation (12). Substituting the results of Equation (13) in Equation (12), we get:

(14) $$H_{Fr}=\underset{q}{\sum }\left\{{\hat{a}}_{0,0}\left(q\right)v\left(q\right)+H.C\right\}|2><2|$$

where,

(15) $$v\left(q\right)=f_{00}\left(q\right)M\left(q\right)$$

where, $f_{00}\equiv f_{l=0,\;m=0}$ and $M\left(q\right)=-\frac{2e}{\pi }\int_{r=0\;}^{r=R}r^{2}dr\;\frac{\sin\left(qr\right)}{qr}\left(\frac{e^{-r^{2}/a_{e}^{2}}}{a_{e}^{3}}-\frac{e^{-r^{2}/a_{h}^{2}}}{a_{h}^{3}}\right)$

Recalling that for l=0, $j_{l=0}\left(q_{n}R\right)=\frac{\sin\left(q_{n}R\right)}{q_{n}R}$ so from Equation (8) we get:

(16) $$q_{n}=\frac{x_{n,0}}{R}=\frac{n\pi }{R}$$

**Figure 1:**
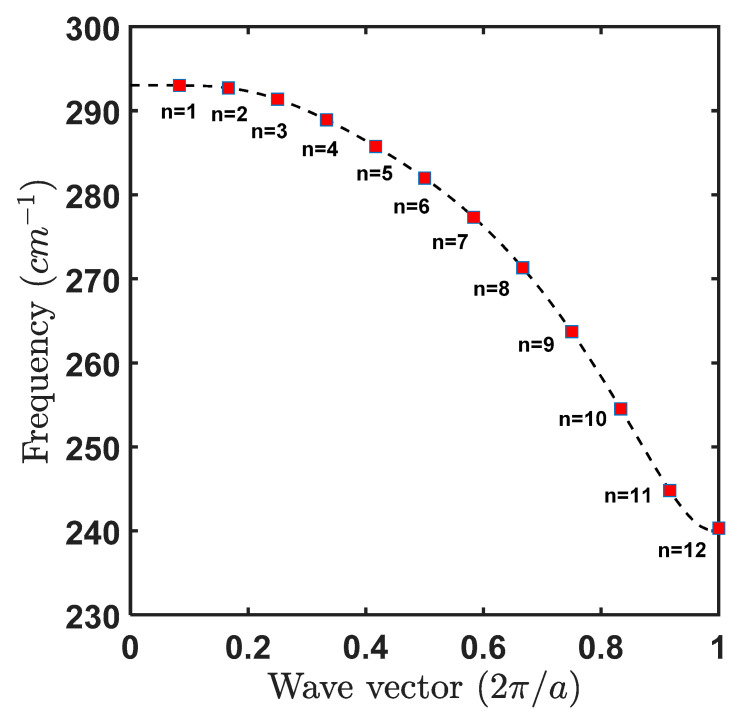
Dispersion relation in GaAs, the dashed lines show for bulk case and the square dots represents the 12 modes obtained for GaAs QD of radius 3.39 nm. In the above figure, a=5.65 Å lattice constant for GaAs.

The discrete phonon dispersion relation obtained for the l=0 mode is plotted below in Figure 1, note that the curve in the dashed line corresponds to the bulk GaAs dispersion relation. 

### 2.3. Confined LO-Phonon-Assisted Exciton Creation for QD Interacting with Classical Light

For the implementation of a QD as a qubit, the QD is subjected to a coherent laser for excitation of the excitonic state. In this section we investigate the confine LO-phonon-assisted creation of excitons in the presence of laser light which can be modelled a classical light. Here, we consider the QD as the two-level system (TLS) consisting of a ground state |1> with a zero-reference energy, and the excitonic state represented by |2>, let the separation of energy $\hbar \omega_{0}$. The theory of a TLS interacting with classical light is well known [18], however, we provide a brief summary. Later in the section, we will introduce the Fröhlich Hamiltonian as a perturbation which triggers the phonon-assisted process. The interacting electric field with the QD can be expressed as:

(17) $$E\left(t\right)=E_{0}\;\hat{\varepsilon }\cos\left(\omega t\right)$$

It is assumed that the interacting electric field has a wavelength much greater than the dimensions of QD, so the spatial dependence in Equation (17) has been dropped. Let the difference between the laser frequency ω and the excitonic resonance frequency $\omega_{0}$ be defined as: detuning, $\Delta =\omega -\omega_{0}$. The interaction of this classical field with the TLS can be expressed as a dipole in the dipole approximation as follows:

(18) $$H_{dot-field}=-d\cdot E$$

where, d is the dipole operator associated with the TLS. The total Hamiltonian which is the summation of TLS bare Hamiltonian $\hbar \omega_{0}\left|2><2\right|$ and $H_{dot-field}$ can be expressed in the Rotating Wave Approximation (RWA) as follows:

(19) $$H_{RWA}=\hbar \left[\begin{array}{cc} 0 & \Omega /2\\ \Omega /2 & -\Delta \end{array}\right]$$

where, Ω is the Rabi frequency defined as:

(20) $$\Omega =-\frac{2\langle 1\left|\hat{\varepsilon }\cdot d\right|2\rangle E_{0}}{\hbar }$$

The Hamiltonian $H_{RWA}$ as in Equation (19) is expressed in the uncoupled bare TLS basis comprising |1> and |2>. The eigen states $H_{RWA}$ are referred to as Dressed basis (which is light + TLS coupled basis) are given as below:

(21) $${|\psi >}_{+}=\sin\theta |1>+\cos\theta |2>$$

(22) $${|\psi >}_{-}=\cos\theta |1>-\sin\theta |2>$$

where, θ is the Stückelberg angle defined as:

(23) $$\tan2\theta =-\frac{\Omega }{\Delta }\;\;$$

**Figure 2:**
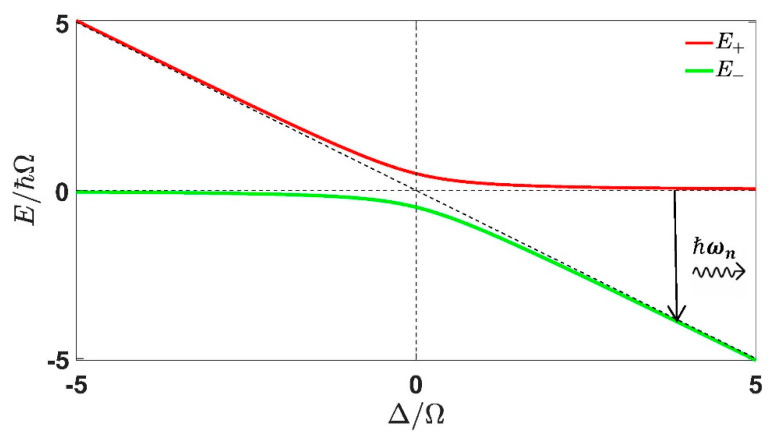
The dressed state energy variation with Δ/Ω is shown. The slant dashed line represents the energy of |2> state with value −ℏΔ and the horizontal dashed line represents the energy of state |1>, when there is no coupling between the bare QD states and the classical light. The solid vertical arrow represents transition from the upper dressed state to lower dressed state when detuning is sufficiently high so that ${|\psi >}_{-}\approx |2>$ and ${|\psi >}_{+}\approx |1>$ and the emitted phonon has the energy $\hbar \Omega^{\Delta }=\hbar \omega_{n}$ (where $\omega_{n}$ is the frequency of confined LO mode of mode n ).

The eigen value of energy of states in Equations (21) and (22) is given as:

(24) $$E_{\pm }=-\frac{\hbar \Delta }{2}\pm \frac{\hbar \sqrt{\Delta^{2}+\Omega^{2}}}{2}=-\frac{\hbar \Delta }{2}\pm \frac{\hbar \Omega^{\Delta }}{2}$$

where, $\Omega^{\Delta }$ is the generalized Rabi frequency. It is clear from Equation (24) that the separation of levels in TLS has now become $\hbar \Omega^{\Delta }$ which was originally $\hbar \omega_{0}$ in the absence of light. From Equation (21), (22) and (23) it is seen that if the detuning is positive such that Δ≫Ω then θ≈π/2, so that $|\psi_{-}\approx |2>$ and $|\psi_{+}\approx |1>$ in such condition phonon-assisted transitions can happen if the exciton decays with an emission of confined LO phonon with energy $\hbar \omega_{n}=\hbar \Omega^{\Delta }$. And since, $\omega_{n}$ is of discrete nature (frequency of confined mode corresponding to wave vector $q_{n}$ as depicted in Figure 1) we can have such phonon-assisted transitions for specific values of detuning at which the energy gap between the dressed state becomes equal to the individual discrete phonon mode of energy $\hbar \omega_{n}$(see Figure 2). We proceed to find such probability per unit time using Fermi’s Golden Rule in the next section.

## 3. Results

### 3.1. Determination of Exciton Creation Rate Assisted by Confined LO Phonon

Using Equations (14), (21) and (22) we can express the exciton—phonon interaction (perturbation) Hamiltonian in the dressed basis as follows:

(25) $$\begin{array}{cc} H_{Fr}=\underset{q,v}{\sum }(v\left(q\right){\hat{a}}_{00}\left(q\right) & +H.C)\;(sin^{2}\theta \;|\psi_{-}><\psi_{-}|\\ & +cos^{2}\theta |\psi_{+}><\psi_{+}|+\frac{1}{2}\sin2\theta |\psi_{-}><\psi_{+}|+\frac{1}{2}\sin2\theta \;|\psi_{+}><\psi_{-}|\;) \end{array}$$

The transition involves change of state from $|\psi_{+},N_{q}>$ to $|\psi_{-},\;N_{q}+1>$, where $N_{q}=\frac{1}{\exp\left(\hbar \omega_{n}/k_{BT}\right)-1}$ is the phonon occupation number of phonon mode with energy $\hbar \omega_{n}$. The transition probability per unit time (or the exciton creation rate) given by:

(26a) $$\frac{1}{\tau }=\frac{2\pi }{\hbar }\underset{q}{\sum }{\left|M_{q}\right|}^{2}\delta \left(E_{-}-E_{+}+\hbar \omega_{n}\right)$$

where,

(26b) $$M_{q}=<N_{q}+1,\psi_{-}|H_{Fr}|\psi_{+},N_{q}>\;$$

So,

(26c) $$M_{q}=\frac{1}{2}\sin2\theta v{\left(q\right)}^{*}\;{\left(N_{q}+1\right)}^{1/2}\;$$

Substituting, $E_{-}-E_{+}=-\hbar \Omega^{\Delta }$ in Equation (26a), we get:

(27) $$\frac{1}{\tau }=\frac{\pi }{2}\sin^{2}2\theta \;J_{ph}\left(\Omega_{n}^{\Delta }\right)\;$$

where, $J_{ph}\left(\Omega^{\Delta }\right)$ is the phonon spectral density as shown below:

(28) $$J_{ph}\left(\Omega_{n}^{\Delta }\right)=\frac{1}{\hbar^{2}}\underset{n}{\sum }{\left|v\left(q\right)\right|}^{2}\delta \left(\Omega^{\Delta }-\omega_{n}\right)\left(N_{q}+1\right)\;$$

To evaluate the phonon spectral density (which is now discrete, hence the generalized Rabi frequency is labelled by subscript n), we substitute $q_{n}=n\pi /R$ in Equation (15) and obtain the now discrete version of M(q) as $M_{n}$ by performing a numerical integration over r, we write the phonon spectral density as below in Equation (29):

(29) $$J_{ph}\left(\Omega_{n}^{\Delta }\right)=\frac{4\pi e^{2}\omega_{LO}}{\hbar R}\left(\frac{1}{\varepsilon_{\infty }}-\frac{1}{\varepsilon_{0}}\right)\left(N_{q}+1\right)\underset{n}{\sum }\frac{M_{n}\delta_{\omega_{n},\Omega_{n}^{\Delta }}}{\omega_{n}}\;$$

**Figure 3:**
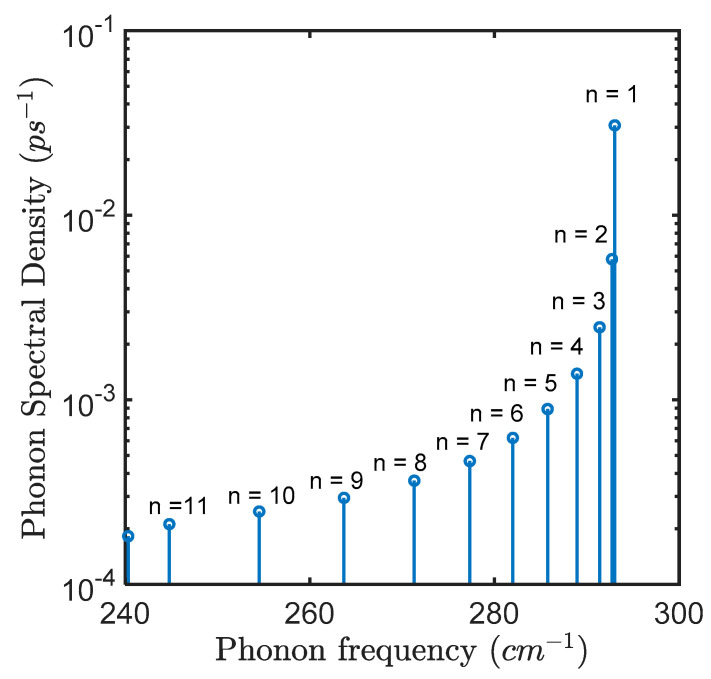
Confined LO mode phonon spectral density for a GaAs quantum dot of radius, R = 3.39 nm at T = 1 K. The discrete spectrum depicts the strength corresponding to each phonon mode contained in the 1st Brillouin zone (total 12 modes), with n=1 having the highest magnitude and is located near the zone center. The n=12 mode appears at 240.32 ${\mathrm{cm}}^{-1}$.

In Figure 3, we plot the discrete phonon spectral density corresponding to all 12 modes (whose discrete dispersion is shown in Figure 1). As we can see that corresponding to n=1 mode which is near the zone center of the Brillouin zone has the highest magnitude of 0.031 ${\mathrm{ps}}^{-1}$ of all the modes. 

**Figure 4:**
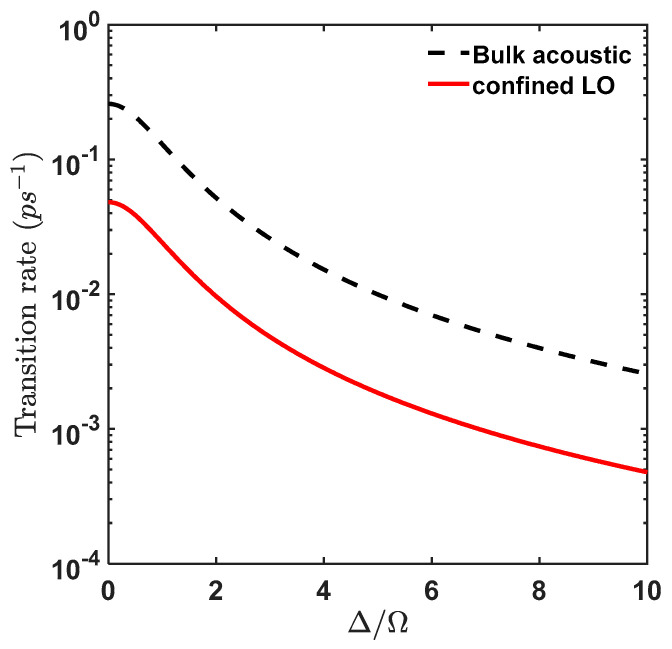
Phonon assisted transition rate between the dressed state for GaAs QD of radius 3.39 nm at T = 1 K. (Red line: confined LO phonon n=1 mode contribution to transition rate, Black line (dashed): bulk acoustic phonon contribution to transition rate).

Now, as we can see from Equation (27), the transition rate is directly proportional to the strength of phonon spectral density, but, from Figure 3 we see that except for n=1 mode the contribution to such transition is negligible. So, for the n=1 mode, Equation (27) can be written as in Equation (30) for which we plot in Figure 4 the transition rate as a function of Δ/Ω:

(30) $$\frac{1}{\tau }=\frac{\pi }{2}\frac{1}{\sqrt{{\left(\Delta /\;\Omega \;\right)}^{2}+1}}\;J_{ph}\left(\omega_{1}\right)\;$$

We would like to point out that, because the phonon frequency $\omega_{1}=\sqrt{\Delta^{2}+\Omega^{2}}$, for any given detuning we can only have a specific value of Ω, hence we choose Δ/Ω to investigate all such possible combinations of allowed Δ and Ω affecting the transition rate.

### 3.2. Exciton Occupancy

Equation (27) represents the transition rate from the lower dressed state to the upper dressed state, the energy gap between the two dressed states is $\hbar \Omega^{\Delta }=\hbar \sqrt{\Omega^{2}+\Delta^{2}}$ (the energy gap depends on applied field strength and detuning). When this gap is equal to one of the confined LO phonon modes then phonon-assisted transitions can result in transitions. And as pointed out in the previous section, that the final state will have more excitonic character if $\frac{\Delta }{\Omega }\gg 1$ when Δ>0 (see Figure 2). Such exciton occupancy in the final state (lower dressed state) is given by:

(31) $${\left|<2|{\psi >}_{-}\right|}^{2}=\sin^{2}\theta =\frac{1}{2}\left(1+\frac{\Delta^{2}}{\Delta^{2}+\Omega^{2}}\right)\;$$

**Figure 5:**
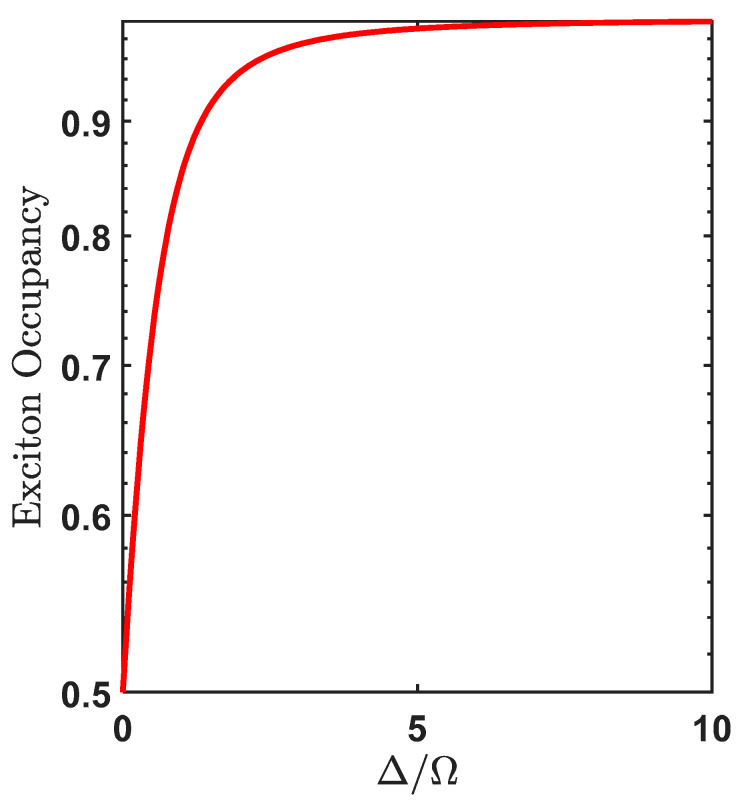
Exciton occupancy (using Equation (32)) in the final state at T = 1 K.

However, in Ref. [19], it was demonstrated that the final exciton occupation taking into account temperature is given by Equation (32) and is plotted in Figure 5:

(32) $$C_{exc}=\frac{1}{2}\left(1+\frac{\Delta }{\Omega^{\Delta }}\tanh\left(\frac{\hbar \Omega^{\Delta }}{2k_{B}T}\right)\right)\;$$

## 4. Discussion

**Figure 6:**
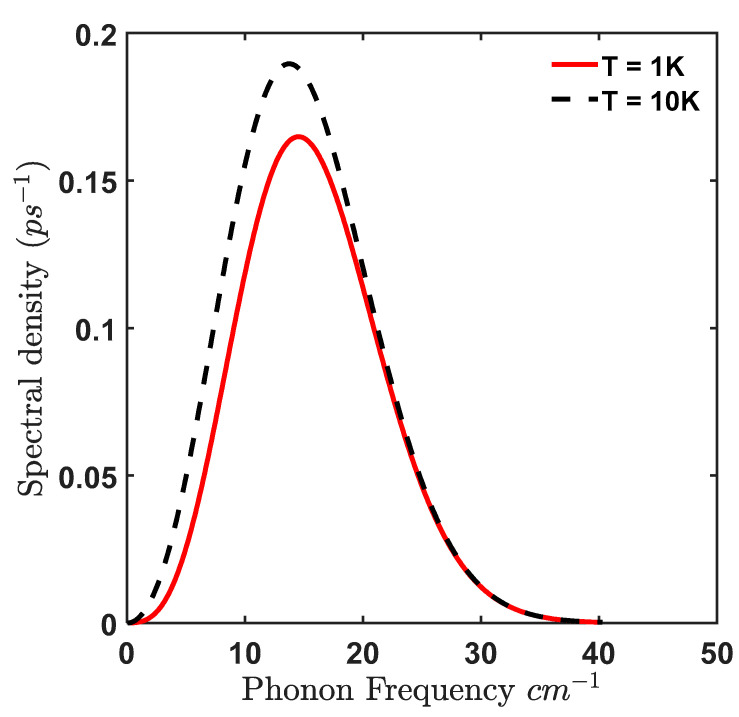
Bulk acoustic phonon spectral density in GaAs at T = 1 K (red) and T = 10 K (black dashed).

It is clear from Figure 4 that the transition rate associated with the confined LO phonon emission decreases with increase in Δ/Ω, however, from Figure 5 it is observed that exciton occupancy increases with Δ/Ω, thus there exists a similar trade-off with confined LO modes as with bulk acoustic phonon modes [20]. At T = 1 K, the maximum transition rate occurs at $\frac{\Delta }{\Omega }=0$ which is of value 0.048 ${\mathrm{ps}}^{-1}$ which corresponds to a relaxation time of 20.83 ps but the exciton occupancy is still 50%. We see that for exciton occupancy corresponding to 80% the corresponding relaxation time is 31.97 ps. Also, we compare our results with transition rate triggered by acoustic phonon. As is well known, there exists very little mismatch between the acoustic properties of the QD material and the barrier material ($Al_{x}Ga_{1-x}As$) in which the QD is embedded so the acoustic confinement is negligible and the bulk acoustic phonon model can be used to evaluate the transition rates leading to the production of excitons. The bulk acoustic phonon density at low temperature is given as:

(33) $$J_{acoustic}\left(\omega \right)=\frac{\omega^{3}}{4\pi^{2}\hbar \rho_{d}c_{LA}^{5}}{\left|\left(D^{e}e^{\frac{-\omega^{2}a_{e}^{2}}{4c_{LA}^{2}}}-D^{h}e^{\frac{-\omega^{2}a_{h}^{2}}{4c_{LA}^{2}}}\right)\right|}^{2}\left(N_{q}+1\right)\;$$

where, $D^{e/h}$ is the electron/hole deformation potential, $\rho_{d}$ is the material density, $c_{LA}$ is the acoustic speed corresponding to longitudinal acoustic (LA) mode. We find that the acoustic spectral density peaks at $\omega =14.61\;{\mathrm{cm}}^{-1}$ with a peak value of 0.1649 ${\mathrm{ps}}^{-1}$ at T = 1 K (see Figure 6). 

Using this value in Equation (30), we find the maximum transition rate for acoustic phonon as a function of Δ/Ω in Figure 5 (black dashed):

Corresponding to an exciton occupancy of 80%, the relaxation time for acoustic phonon at T = 1 K is 5.95 ps, hence it is 5.37 times less than the confined LO mode at the same temperature. However, the average transition rate for confined LO mode for exciton occupancy above 80% for temperature T≤10 K is 0.042 ${\mathrm{ps}}^{-1}$ (that corresponds to relaxation time of 23.8 ps) whereas for acoustic phonon mode, the average transition rate for T≤10 K is 0.24 ${\mathrm{ps}}^{-1}$ (that corresponds to relaxation time of 4.17 ps). Hence, the exciton creation rate attributed to confined LO phonon is 5.7 times (~6 times) slower than acoustic phonon.

## 5. Conclusions

We investigated the role of confined LO phonons in GaAs QD taking a radius of 3.39 nm as representative of a strong confinement limit. We found that phonon relaxation time corresponding to confined LO mode is approximately 6 times higher than acoustic phonon at low temperatures, T≤10 K. It is clear that acoustic modes are the dominant phonon relaxation mechanism, however at low temperatures (≤10 K), the confined LO phonon cannot be neglected at all, hence it must be taken into account. Moreover, our results provide the theoretical basis for exciton preparation based of the use of optical-phonon-assisted processes rather than acoustic-phonon processes.

## Data Availability

Not applicable.
